# Computational drug repurposing reveals Alectinib as a potential lead targeting Cathepsin S for therapeutic developments against cancer and chronic pain

**DOI:** 10.3389/fbinf.2025.1666573

**Published:** 2025-09-24

**Authors:** Mohammed Alrouji, Mohammed S. Alshammari, Sharif Alhajlah, Syed Tasqeeruddin, Khuzin Dinislam, Anas Shamsi, Saleha Anwar

**Affiliations:** 1 Department of Medical Laboratories, College of Applied Medical Sciences, Shaqra University, Shaqra, Saudi Arabia; 2 Department of Clinical Laboratory Sciences, College of Applied Medical Sciences, Shaqra University, Shaqra, Saudi Arabia; 3 Department of Pharmaceutical Chemistry, College of Pharmacy, King Khalid University, Abha, Saudi Arabia; 4 Department of General Chemistry, Bashkir State Medical University, Ufa, Republic of Bashkortostan, Russia; 5 Centre of Medical and Bio-allied Health Sciences Research, Ajman University, Ajman, United Arab Emirates

**Keywords:** cathepsin S, cancer, chronic pain, drug repurposing, small molecule inhibitors, virtual screening

## Abstract

Cathepsin S (CathS) is a cysteine protease known to play a role in extracellular matrix (ECM) re-modelling, antigen presentation, immune cells polarisation, and cancer progression and chronic pain pathophysiology. CathS also causes an immunosuppressive environment in solid tumors and is involved in nociceptive signaling. Although several small-molecule inhibitors with favorable *in vivo* properties have been developed, their clinical utility is limited due to resistance, off-target effects, and suboptimal efficacy. Therefore, alternative therapeutic strategies are urgently needed. In the present study, we utilized an integrated virtual screening protocol to screen 3,500 commercially available FDA-approved drug molecules from DrugBank against the CathS crystal structure, based on which drug-likeness profile and interaction studies were performed to filter putative candidates. Alectinib was found to be a top hit and had significant interactions with the important active-site residues His278 and Cys139. PASS predictions suggested relevant anticancer and anti-pain activities for Alectinib in reference to the control inhibitor Q1N. Later, 500-ns molecular dynamics simulations under the CHARMM36 condition revealed that the CathS-Alectinib complex maintained its structural stability, as indicated by conformational parameters, hydrogen-bond persistence, and essential dynamics analyses. Further MM-PBSA calculations also confirmed a favorable binding free energy (Δ*G* –20.16 ± 2.59 kcal/mol) dominated by the van der Waals and electrostatic contributions. These computational findings suggest that Alectinib may have potential as a repurposed CathS inhibitor, warranting further experimental testing in relevant cancer and chronic pain models. Notably, these results are based solely on computational analysis and require empirical validation.

## 1 Introduction

Proteases are a large family of enzymes that mediate proteolytic cleavage of proteins and govern a wide variety of biological processes, from intracellular protein decay to extracellular matrix reshaping to immune signaling (Verhamme et al., 2018). Of the cysteine cathepsins, Cathepsin S (CathS) is particularly remarkable owing to its distinct feature in retaining catalytic activity at neutral pH, allowing it to operate intracellularly as well as extracellularly (Vidak et al., 2019). Under steady-state conditions, CathS-induced processing of the class II-associated invariant chain on major histocompatibility complex (MHC) has a critical role for antigen presentation to CD4^+^ T cells (Costantino et al., 2009). Concurrently, the regulated extracellular activity of CathS allows selective degradation of matrix proteins like collagen and elastin, facilitating normal tissue remodeling and immune cell traffic (Fonović and Turk, 2014).

Nevertheless, abnormal CathS expression and activity have been linked with a variety of diseases (Wilkinson et al., 2015). In human oncology, the tumor microenvironment of breast, colorectal, lung, and pancreatic cancer is often characterized by high levels of CathS (Small et al., 2013). In this scenario, CathS-mediated degradation of the ECM not only creates pathways for cancer cell invasion and metastatic spread but also modulates the cytokine and chemokine milieu by favoring an immunosuppressive microenvironment, thereby thwarting anti-tumor immune surveillance (Liu et al., 2023). Particularly, CXCL12 can be cleaved and inactivated by CathS, thus inducing the suppression of effector T cell infiltration, but in the meantime generating fragments that attract regulatory T cells and myeloid-derived suppressor cells (Jakoš et al., 2019). High CathS expression is clinically associated with tumor aggressive phenotypes, poor patient survival, and resistance to conventional chemotherapy as well as immunotherapy (Wilkinson et al., 2015; Liang et al., 2023).

Besides cancer, CathS is becoming better known as a central player in chronic pain (Montague-Cardoso and Malcangio, 2020). Mechanical injury or inflammatory challenges to the peripheral nerve induce microglial activation in the spinal cord and subsequent release of CathS into the extracellular space (Clark and Malcangio, 2012). CathS cleaves protease-activated receptor 2 (PAR2) on nociceptive neurons, leading to signal transduction pathways contributing to neuron excitability and central sensitization (Zhao et al., 2014). Similarly, in animal models of neuropathic and inflammatory pain, pharmacological inhibition or genetic deletion of CathS reduces pain behaviors, thus highlighting CathS as a potential target for analgesics (Clark et al., 2007). In addition, an increased level of CathS activity was found in cerebrospinal fluid and serum of patients suffering from painful neuropathies, indicating translational importance (Montague-Cardoso and Malcangio, 2020).

However, despite accumulating evidence for a potent therapeutic role of CathS in both oncology and pain, attempts to develop specific, drug-like inhibitors have been challenging (Ajani et al., 2024). The early generation inhibitors were predominantly peptide-derived and employed electrophilic warheads like epoxysuccinates, vinyl sulfones, or acyloxymethyl ketones to synthetically modify the catalytic cysteine covalently (Mehrtens, 2007; Wilkinson et al., 2015). However, these inhibitors demonstrated low-nanomolar potency *in vitro*; however, they were not cell permeable, were rapidly degraded by proteases, and had low oral bioavailability (Ajani et al., 2024). Structural-based medicinal chemistry approach provided nonpeptidic scaffolds (e.g., thiosemicarbazones, nitriles, and heterocyclic aldehydes) that enhanced pharmacokinetic properties, but many of the inhibitors led to off-target inhibition of close homologs of cathepsins (e.g., Cathepsin K, L, or B) with resulting dose-limiting toxicities in preclinical models (Frizler et al., 2010). Furthermore, penetration into tissue has proven difficult, especially in tumor stroma or inflamed neural tissue, and often requires high systemic exposure raising concerns with respect to safety and tolerability.

Considering these continuing challenges, the idea of drug repurposing has become an appealing alternative to traditional *de novo* discovery (Pushpakom et al., 2019). Using already established pharmacological and toxicological information on approved drugs, therapy revitalization can significantly accelerate the pathway from target discovery to the clinic, decrease development expenses, and lessen the safety concerns (Parvathaneni et al., 2019). Over the past decade, advances in computational chemistry and structural biology have enabled the virtual screening of approved drug libraries against high-resolution target structures (Shamsi et al., 2024a; Shamsi et al., 2024b). This approach allows for the rapid identification of candidate compounds based on binding affinity, interaction fingerprints, and pharmacophore matching. These have led to success stories in a wide range of disease areas (e.g., repositioning known antivirals as kinase inhibitors and discovering antipsychotics have unexpected anti-fibrotic activity), highlighting the strength of *in silico* repurposing in revealing unknown activities of existing drugs (Mottini et al., 2021).

Repurposing is of particular interest for CathS for therapeutic developments. Many oncology drugs have physicochemical properties that facilitate tumor penetration and retention, and some CNS drugs have been designed to traverse the blood–brain barrier, which is favorable for spinal microglia targeting in the context of neuropathic pain (Kuthati et al., 2020; Alghamri et al., 2021). Many approved drugs also contain scaffolds suitable for synthetic modification on the rapid time scales necessary for hit to lead optimization, following detection of primary hits (Verma and Pathak, 2022). Crucially, as the safety, metabolism, and formulation behavior of these compounds are already known, the most promising repurposed adjuncts can relatively rapidly advance into phase II or phase III trials for new indications (Schein, 2020).

The current study attempts to create a comprehensive virtual screening cascade to discover FDA-approved drugs with as yet unappreciated inhibitory activity towards CathS. The approach begins with molecular docking of an *in silico* curated library of approved drugs into the active site of CathS using a high-resolution crystal structure. Top-scoring hits are then filtered through drug-likeness and ADMET predictors to ensure favorable pharmacokinetic and safety profiles. Following this, ligand–protein interaction fingerprints are analyzed to assess the engagement of key catalytic residues and subsites. Finally, the study lays the groundwork for subsequent molecular dynamics and free-energy calculations to validate the stability and affinity of the predicted binding interactions. By using a structure-based docking approach, integrated with cheminformatics and physicochemical filtering, we hope to quickly identify repositioning candidates presenting a potentially appropriate equilibrium of potency, selectivity, and tissue distribution properties, applicable to cancer and pain indications.

Ultimately, this study identifies new CathS inhibitors among the approved drugs and suggests a broadly applicable repurposing pipeline applicable for other protease targets. The successful pursuit of this goal could expedite the clinical translation of CathS-targeted therapies, providing new opportunities for the treatment of tumor metastasis, chronic pain, and immune-mediated diseases. Furthermore, the discovery of dual-purpose molecules, acting upon CathS in both oncology and neuropathic pain contexts, might pave the way for novel multi-indication clinical trials, fitting into the recent development of multitargeted drugs.

## 2 Materials and methods

### 2.1 Molecular docking screening

We screened compounds with high affinity to CathS using structure-based docking. The crystal structure of the enzyme was downloaded from the RCSB Protein Data Bank (Bank, 1971) (PDB ID: 6YYR) and processed by rebuilding the missing residues, protonating the polar groups, and assigning the proper atom types. A DrugBank (Knox et al., 2024) FDA-approved drugs library was then prepared for docking with InstaDock v1.2 (Mohammad et al., 2021) and AutoDock Tools (Morris et al., 2009). 62 × 52 × 66Å docking grids were placed at the center of InstaDock coordinates (21.428, 5.236, 14.12), and binding studies were performed via both InstaDock and MGL AutoDock. Pose outputs were visualized and analyzed by PyMOL (DeLano, 2002) and Discovery Studio Visualizer (Visualizer, 2005), and compounds were ranked by predicted binding energy. The best candidates were chosen for detailed computational estimation.

### 2.2 Drug profiling, PASS, and interaction analyses

The selected compounds from the docking screening were further screened through drug profiling to test their pharmacological and therapeutic actions. The identified molecules from the docking screening were then evaluated for their drug-like properties and pharmacological activities. The biological activity of selected compounds was estimated using the PASS (Prediction of Activity Spectra for Substances) online tool (Filimonov et al., 2014). This tool can infer the biological activity of a molecule based on its structural similarity to known bioactive compounds in a large training set. It focuses on the prediction in ‘probability of activity (Pa)’ and “probability of inactivity (Pi)”, the higher the Pa value, the higher is the likelihood of a particular predicted biological activity associated with the compound. After the PASS analysis, the binding modes and geometry of the CathS active site-bound screened compounds were estimated. Polar contacts computed in PyMOL for ligand interaction with CathS provided information about the binding orientations. To study the molecular interactions in more detail, Discovery Studio Visualizer was used, which specifically focused on the binding pocket of CathS. Hit compounds that showed robust interactions with the active site, particularly those that were situated at the active site interface, were then selected for further analysis.

### 2.3 MD simulations

The processed protein and protein-ligand structures were subjected to all-atom MD simulations for 500 ns. One lead compound and one reference compound were selected for MD simulations based on the docking scores, molecular interactions, and potential biological activities. The simulations were performed under near-physiological conditions using GROMACS version 2022 (Van Der Spoel et al., 2005). System setup employed the CHARMM36-July2022 force field (Huang and MacKerell Jr, 2013). All systems were solvated in a cubic box of TIP3P water (Mark and Nilsson, 2001), with at least a 10 Å buffer from any protein atom to the box edge. The box was neutralized by adding counterions to achieve ∼0.15 M ionic strength. The ligand topologies were obtained with the CGenFF server (Zhu, 2019). The systems were energy minimized over 50,000 steps and subjected to a two-stage equilibration: first in the NPT ensemble (298 K, 1 atm) for 1 ns, then in the NVT ensemble for 1 ns. Periodic boundary conditions were employed throughout the simulations. All bonds involving hydrogen atoms were constrained (using the LINCS algorithm), allowing a 2 fs time step. We used a multi-time stepping algorithm with bonded interactions being updated every time step and short-range and long-range electrostatic interactions calculated every two time steps. Long-range electrostatics were treated using the Particle Mesh Ewald (PME) method. This work was performed on a Dell workstation with Ubuntu, and the final production simulation was performed for 500 ns. Post-simulation analysis of the trajectories was performed using the built-in analysis tools of GROMACS.

### 2.4 Principal component and free energy landscape analyses

In order to reduce the complexity of the MD trajectory data and isolate the global collective and local atomic motions, we carried out principal component analysis (PCA) (Tharwat, 2016). A limited number of dominant PCs were selected to represent the largest conformational changes of the protein-ligand complex. PCA was performed by *gmx covar* and *gmx anaeig* tools in GROMACS. The *gmx covar* tool was used to calculate the covariance matrix, which describes the correlated movements of backbone atoms of the proteins. The eigenvectors serve as motion directions and the eigenvalues as corresponding magnitudes to describe dynamic behavior and atomic contributions of the complex. To illustrate the motions, a 2D projection of the trajectory was obtained by projecting the first and second principal components onto 2D with *gmx anaeig*. Free energy landscapes (FELs) (Maisuradze et al., 2010) were also generated to evaluate the folding behavior or probable aggregation of the protein−ligand complex. Free energy calculations allow the determination of the system’s overall structural stability.

### 2.5 MM-PBSA calculations

The molecular mechanics Poisson-Boltzmann surface area (MM/PBSA) method (*gmx_MMPBSA*), coupling GROMACS simulation data with a solver-based solvation analysis, was used for calculating binding free energies (Genheden and Ryde, 2015; Valdés-Tresanco et al., 2021). For each system, a 50 ns segment of the end region of the MD trajectory was chosen, with snapshots recorded every 10 ps. The overall binding free energy was calculated by adding the relevant contributions of vdW (van der Waals), electrostatics, polar solvation energy and non-polar solvation using solvent accessible surfac area (SASA). The binding free energy ( ${∆G}_{binding}$ ) was calculated using the standard thermodynamic cycle as follows:

$${∆G}_{binding}=G_{complex}\;–\;\left(\;G_{receptor}+G_{ligand}\;\right)$$

Each term in the equation represents the free energy associated with the respective molecular components. Furthermore, per-residue energy decomposition was carried out using the Python-based analysis tools bundled with the *gmx_MMPBSA* package.

## 3 Results and discussion

### 3.1 Molecular docking screening

Molecular docking is a computer-based approach used to examine the way and orientation in which ligands interact with the active site of the target protein (Shamsi et al., 2019). For the analysis, a 3500 FDA-approved drug data set was obtained from the DrugBank database (https://go.drugbank.com/). These compounds were virtually screened on CathS with InstaDock, looking for potential high-affinity binders. The top 10 compounds based on binding scores with CathS were selected after docking (Table 1). From the docking results, it could be seen that these hit compounds had good binding affinities with docking scores from −11.0 to −12.3 kcal/mol. These are scores for stronger (lower (more negative) scores are generally indicative of a stronger) ligand-protein interactions. The docking score of the reference inhibitor Q1N was found to be −9.7 kcal/mol, and all top 10 compounds showed better binding affinity than Q1N. This is explained by the results, which indicate that the studied compounds might be powerful competitive inhibitors of CathS and might provide an incentive for their further exploration as a future therapeutic agent.

**TABLE 1:** List of screened hits against CathS and their docking parameters.

S. no.	Drug	Binding affinity (kcal/mol)	p*Ki*	Ligand efficiency (kcal/mol/non-H atom)	Torsional energy
1	Tasosartan	−12.3	9.02	0.3968	1.2452
2	Bagrosin	−12.1	8.87	0.55	0.3113
3	Bisantrene	−11.4	8.36	0.38	1.8678
4	Lumacaftor	−11.4	8.36	0.3455	1.8678
5	Tadalafil	−11.1	8.14	0.3828	0.3113
6	Adapalene	−11.1	8.14	0.3581	1.5565
7	Eltrombopag	−11.1	8.14	0.3364	2.1791
8	Pimozide	−11.1	8.14	0.3265	2.1791
9	Alprazolam	−11.0	8.07	0.5	0.3113
10	Alectinib	−11.0	8.07	0.3056	0.9339
11	Q1N	−9.7	7.11	0.2939	2.8017

### 3.2 Drug profiling and PASS analysis

The hit molecules obtained through docking screening were subjected to further assessment in terms of their drug-like features and their biological activities that may be related to CathS-driven diseases. From the 10 selected compounds, Alectinib emerges as a good candidate according to the druggability profile and activity prediction. Alectinib was selected due to its drug likeness, known kinase inhibitory activity, and FDA approval for anti-cancer therapy, as it is connected well with CathS-relevant pathologies (Supplementary Table S1). Notably, although some other compounds (e.g., Tasosartan and Bagrosin) had marginally stronger docking scores, their primary clinical uses (e.g., as an antihypertensive and an antitumor agent, respectively) are less directly related to the cancer/pain indications targeted by CathS inhibition. In contrast, is an ALK-targeted anticancer drug with a well-characterized safety profile, making it a more relevant and promising repurposing candidate for CathS-associated cancer and pain contexts. The PASS server (Filimonov et al., 2014) was used to explore possible biological activities of molecules docking into the target protein. PASS analysis also indicated that Alectinib was a highly active anticancer (Table 2). Alectinib had somewhat higher probabilities of activity (Pa) than inactivity (Pi), especially for anticancer, antimigraine, nootropic, and chronic pain (Pa 0,140–0,533). These results reveal the repurposable potential of Alectinib as a promising repurposed CathS inhibitor. The reference compound Q1N was also evaluated for its biological activity with a Pa value between 0,077 and 0,369, lower than Alectinib drug. An additional pharmacological consideration is Alectinib’s favorable pharmacokinetic profile, particularly its ability to cross the blood–brain barrier (BBB), which is clinically relevant for neuropathic pain indications. This property supports its candidacy for repurposing where CNS penetration is necessary, although its activity against CathS in the CNS remains to be experimentally validated.

**TABLE 2:** PASS analysis of the selected molecules with their predicted activity.

S. no.	Drug	Pa	Pi	Activity
1	Alectinib	0,533	0,049	Neurotransmitter uptake inhibitor
		0,327	0,073	MAP3K5 inhibitor
		0,349	0,126	Antineoplastic
		0,364	0,253	Nootropic
		0,140	0,085	Antimigraine
2	Q1N	0,369	0,080	Fibroblast growth factor agonist
		0,181	0,057	Antineoplastic
		0,144	0,094	Liver fibrosis treatment
		0,048	0,018	Matrix metalloproteinase 1 inhibitor
		0,077	0,068	Multidrug resistance-associated protein inhibitor

### 3.3 Interaction analysis

In the past 20 years, molecular docking has become a key procedure in contemporary *in silico* drug discovery owing to its ability to predict the interactions of small molecules with protein targets at the atomic level. This approach allows for the exploration of how potential therapeutics can bind to the active site on a particular protein and convey a better understanding of the biochemical mechanisms underlying such binding. After docking, complexes are analyzed to pick out the most promising candidates for further exploration. The binding affinity of each ligand is predicted by the computed interaction energies, and the compounds are then sorted. Further analysis of the docked conformations may facilitate the identification of the key molecular interactions (e.g., hydrogen bonding, hydrophobic contacts, and electrostatic contacts) that are responsible for the stability and specificity of ligand binding to the receptor. These findings contribute to a deeper insight into the potential mechanism of action of the ligand and provide rational design and structural optimization in the later development of new drugs.

**FIGURE 1:**
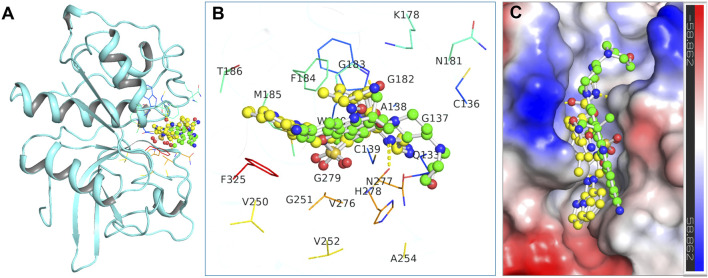
Binding interactions of the docked compounds with Cathepsin S (CathS). **(A)** Docked view showing CathS complexed with Alectinib (green) and Q1N (yellow). **(B)** Magnified view of the key residues in the CathS active site interacting with Alectinib and Q1N. **(C)** Surface representation of the CathS binding pocket with bound Alectinib and Q1N, highlighting how both ligands occupy the catalytic cleft.

In this context, Alectinib was examined for its docked interactions with CathS (Figure 1). Q1N was used as a reference compound for comparison. As shown in Figure 1A, Alectinib interacts with several crucial residues in the catalytic site of CathS and forms various close interactions such as hydrogen bonds and hydrophobic contacts. The analysis indicated that Alectinib and the reference ligand form significant interactions with crucial active site residues, including the active-site residues His278 and Cys139, and various other binding site residues (Figure 1B), suggesting a potentially favorable binding mode. In addition, both compounds fitted tightly into CathS’ binding pocket (Figure 1C). These results indicate that Alectinib fits into the catalytic site of CathS and could have favorable interactions. Taken together, the findings support further investigation of Alectinib as a potential repurposing candidate for CathS inhibition.

**FIGURE 2:**
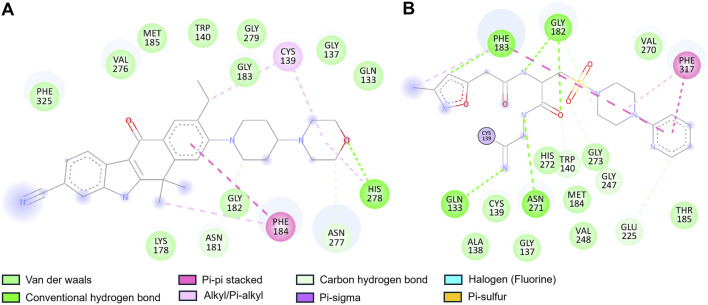
Two-dimensional interaction diagrams of Alectinib and Q1N with Cathepsin S. **(A)** Alectinib interaction map showing multiple stabilizing contacts with active-site residues. **(B)** Q1N interaction map depicting hydrogen bonds with the CathS residues. Various colors highlight different types of interactions (e.g., hydrogen bonds, hydrophobic contacts) between the ligand and protein.

Furthermore, a detailed interaction analysis between Alectinib and Q1N with the CathS was analyzed using Discovery Studio Visualizer. The 2D interaction map of Alectinib is shown in Figure 2, and we can observe different types of interactions with important residues. Alectinib intercalated into residues within the dimer interface and formed several interactions similar to Q1N (Figures 2A,B). Alectinib engaged in several interaction patterns, such as hydrogen bond formation (Asn181, Asn277, His278), π–π stacking (Phe184), alkyl/π-alkyl (Cys139, Phe184, His278), and van der Waals contacts (Gln133, Gly137, Trp140, Lys178, Gly182, Gly183, Met185, Val276, Gly279, Phe325) (Figure 2A). At the same time, Q1N was involved in several interactions such as hydrogen bonds (Gln133, Trp140, Gly182, Phe183, Glu225, Gly247 and Asn271), π–π stacking (Phe183, Phe317), alkyl/π-alkyl (Phe183, Phe317) and van der waals contact (Gly137, Ala138, Cys139, Met184, Thr185, His272, Gly273, Val248 and Val270) (Figure 2B). Of note, six residues (Gln133, Gly137, Cys139, Trp140, Phe184, and Met185) were common to Alectinib and Q1N, implying to have an overlapping binding site in the CathS. His278 and Cys139, which were implicated in Alectinib binding, which occurred at the active site, whereas only Cys139 took part in interactions at the active site with Q1N. These data indicated that Alectinib can bind to CathS tightly and interact with the critical active site residues, which implied that CathS could be a potential target of Alectinib in cancer therapy. These observations were further studied in all-atom 500 ns MD simulations as discussed in the ensuing sections.

### 3.4 MD simulations

MD simulation is a powerful tool to investigate the behavior of macromolecules under physiological conditions (Shukla and Tripathi, 2021). In this study, we performed a 500 ns MD simulation of the CathS before and after drug binding. Post analysis was done using GROMACS' inbuilt tools, and figures were generated by the XMGrace tool (Turner, 2005). Initially, to ascertain the binding stability of the complexes potential energy was calculated. Potential energy (kJ/mol) of CathS, CathS-Q1N, and CathS-Alectinib complex were −590618, −407925, and −407370, respectively. CathS has shown highly negative energy, which indicates more stability of the native fold of the protein, while both complexes show similar amounts of energy, which is less negative or higher than CathS, and comparatively suggest that the bound complexes were stable.

#### 3.4.1 RMSD and RMSF analysis

**FIGURE 3:**
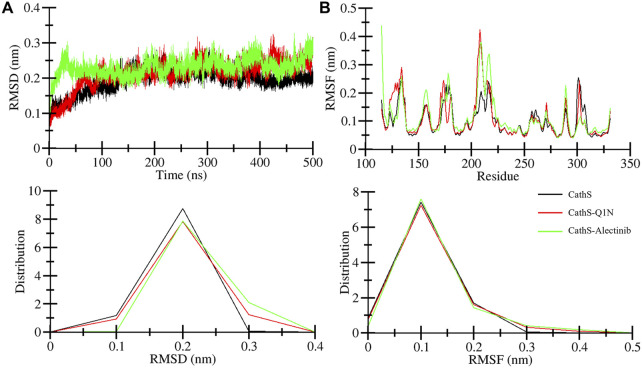
Stability assessment of CathS, CathS-Q1N, and CathS-Alectinib complex by analyzing **(A)** RMSD plots over 500 ns showing backbone stability of CathS, CathS-Q1N, and CathS-Alectinib, **(B)** RMSF plots indicating per-residue flexibility during simulations. Lower panels show distribution plots of RMSD and RMSF values across trajectories. Both complexes exhibit stable trajectories with limited fluctuations, consistent with sustained ligand binding.

The association of systems stability through root mean square deviation (RMSD) analysis is closely studied and proven (Maiorov and Crippen, 1994). RMSD is a well-recognized approach to observe system stability in a given time frame and associated factors such as ligand binding and mutations (Mohammad et al., 2020). Figure 3A displays stability assessment through the RMSD plot generated for a 500 ns simulation. The average RMSD values for CathS, CathS-Q1N, and CathS-Alectinib complex were 0.19 nm, 0.21 nm, and 0.23 nm, respectively (Table 3). The trajectory of the CathS and CathS-Q1N complex was found to be similar through the simulation run. The trajectory of the CathS-Alectinib complex reflects initial fluctuation till around 80 ns, then the trajectory pattern remained steady with some random minor fluctuations throughout the MD run. The distribution plot of the CathS, CathS-Q1N, and CathS-Alectinib complex reflects minimal changes among the systems’ trajectories. Overall, RMSD analysis reflects that both complexes were in stable form with reference to the CathS during the 500 ns simulations.

**TABLE 3:** Mean values of the analyzed parameters for CathS, CathS-Q1N, and CathS-Alectinib complex.

Systems	RMSD (nm)	RMSF (nm)	*R*g (nm)	SASA (nm^2^)	Intra H-bonds
CathS	0.19	0.09	1.6	107.4	150
CathS-Q1N	0.21	0.10	1.7	113.8	144
CathS-Alectinib	0.23	0.11	1.7	110.4	147

Root mean square fluctuation (RMSF) is another well-studied approach to measure changes in individual amino acids’ behavior during the simulations. RMSF estimates the fluctuation range of the amino acid residues with a function for time in association with given factors. Residual fluctuation for CathS, CathS-Q1N, and CathS-Alectinib complex is given in Figure 3B. The average RMSF value for CathS, CathS-Q1N, and CathS-Alectinib complex was calculated as 0.09 nm, 0.10 nm, and 0.11 nm, respectively (Table 3). In reference to CathS and CathS-Q1N complex, CathS-Alectinib complex shows a similar pattern. The reference CathS-Q1N complex shows a higher fluctuation range between 204 and 210 amino acid residues, and relatively CathS-Alectinib complex also shows fluctuations between 206 and 218 residues. Apart from these fluctuations, some minor fluctuations were also seen, which do not show any major impact on complex stability. In addition, the distribution plot was also generated to observe the pattern of residue distribution range; the plot shows an overlapped pattern of distribution for CathS, CathS-Q1N, and CathS-Alectinib complex. Consequently, the result shows that complexes were in a stable state after drug binding.

#### 3.4.2 Rg and SASA analysis

**FIGURE 4:**
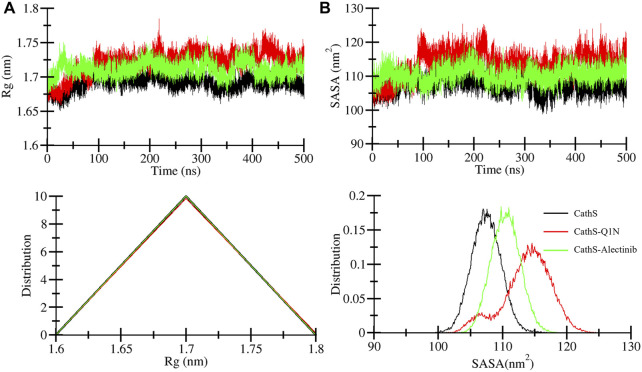
Compactness and solvent exposure of CathS and ligand-bound complexes. **(A)** Radius of gyration (*R*g) plots show protein compactness across 500 ns simulations. **(B)** Solvent-accessible surface area (SASA) plots illustrate changes in solvent exposure of protein surfaces. Lower panels display distribution plots of Rg and SASA values. Alectinib binding maintains CathS compactness and stability, with values comparable to or lower than the Q1N complex.

The radius of gyration (*R*g) indicates the compactness of the structure under the given factors and physiological conditions (Lobanov et al., 2008). We examined *R*g to analyze the compactness of CathS after ligand binding for 500 ns. Figure 4A shows the plot of CathS, CathS-Q1N, and CathS-Alectinib complex in different colors. The average *R*g value for CathS was 1.6 nm, and for both CathS-Q1N and CathS-Alectinib complex *R*g value was 1.7 nm (Table 3). The CathS reflects the most stable reference, which shows a lower pattern of the *R*g trajectory during the 500 ns simulation. The reference CathS-Q1N complex reflects a higher fluctuation range compared to. CathS-Alectinib complex was in a steady pattern throughout the simulation and showed more compactness than the reference CathS-Q1N complex. Moreover, the *R*g distribution plot shows similar patterns, not even minor changes in the *R*g distribution of CathS, CathS-Q1N, and CathS-Alectinib complex. The comparative findings suggest that the CathS-Alectinib complex was in compact form after a 500 ns simulation.

Solvent accessible surface area (SASA) is a part of the protein that can be accessible to the surrounding solvent during the simulations (Durham et al., 2009). The SASA approach is used to measure folding and unfolding patterns of the molecules; if SASA increases, it reflects molecules stability or *vice versa*. Average SASA values for CathS, CathS-Q1N, and CathS-Alectinib complex were 107.4 nm^2^, 113.8 nm^2^, and 110.4 nm^2^, respectively (Table 3). In Figure 4B, the red plot of reference CathS-Q1N complex shows a higher SASA value after 85 ns till the end of the simulation. The CathS plot showed in decrease, which clearly defines a stable form, and the plot of CathS-Alectinib complex was in a very steady and stable state. The Distribution plot also indicates various SASA values in which the CathS-Q1N complex achieved higher. Overall, SASA findings show an association with the *R*g result, which states that the complex folded well and is observed in a stable state.

#### 3.4.3 Hydrogen bonds analysis

**FIGURE 5:**
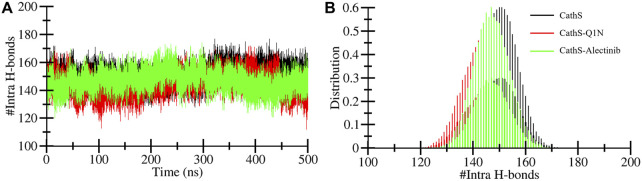
Intramolecular hydrogen bond dynamics of CathS and ligand-bound complexes. **(A)** Time evolution of intramolecular hydrogen bonds in CathS, CathS-Q1N, and CathS-Alectinib. **(B)** Distribution plots of intramolecular hydrogen bond counts. Both complexes retain a stable network of internal hydrogen bonds, suggesting that the global structural integrity of CathS is preserved upon ligand binding.

Intramolecular hydrogen bonds are formed with protein molecules, which play a crucial role in maintaining structural shape, three-dimensional conformation, stability, and enhancing function (Hubbard and Haider, 2010). A higher number of bonds is closely associated with the stability of the structure, while breaking bonds tends to unfold or destabilize the structure. The average bond formation for CathS, CathS-Q1N, and CathS-Alectinib complexes is 150, 144, and 147, respectively (Table 3). Figure 5 displays the plot of CathS, CathS-Q1N, and CathS-Alectinib complex, which reflects hydrogen bonds over time. After initial adjustment, the CathS and CathS-Q1N complex shows distortion of hydrogen bonds to 300, afterward, hydrogen bonds increase (Figure 5A). CathS-Alectinib complex shows minor fluctuations in bond formation, between 200 ns and 300 ns, the number of hydrogen bonds was at a higher peak; later, the bonds were decreased, which might be due to the binding adjustment. Moreover, distribution also reflects variation of hydrogen bonds; collectively, the result indicates stable complex was seen (Figure 5B).

**FIGURE 6:**
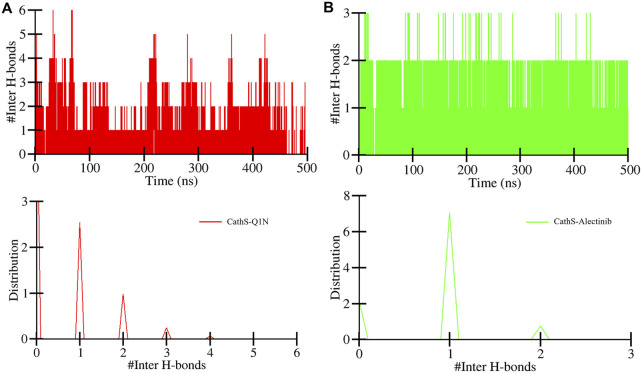
Intermolecular hydrogen bond dynamics between CathS and ligands. **(A)** Time evolution of hydrogen bonds between CathS and Q1N. **(B)** Time evolution of hydrogen bonds between CathS and Alectinib. Lower panels show distributions of hydrogen bond counts. Alectinib consistently maintains up to three hydrogen bonds throughout the simulation, with two being stable over the entire 500 ns trajectory, indicating persistent protein–ligand recognition.

Intermolecular hydrogen bonds formed between the ligand and protein play a crucial role in structure recognition and stability (Bitencourt-Ferreira et al., 2019). The intermolecular plot in Figure 6 shows the bond formation during the 500 MD run. CathS-Q1N complex indicates a maximum of 6 bonds, while CathS-Alectinib complex indicates a maximum of 3 hydrogen bonds (Figure 6A). The bond consistency between the CathS-Q1N complex was lower, while in the CathS-Alectinib complex, 2 hydrogen bonds were consistent throughout the MD run (Figure 6B). In the distribution plot, CathS-Alectinib comparatively indicates a higher distribution of the bonds during the 500 ns MD run. Relatively, these findings suggest that the CathS-Alectinib complex was in a stable form.

#### 3.4.4 Secondary structure analysis

**FIGURE 7:**
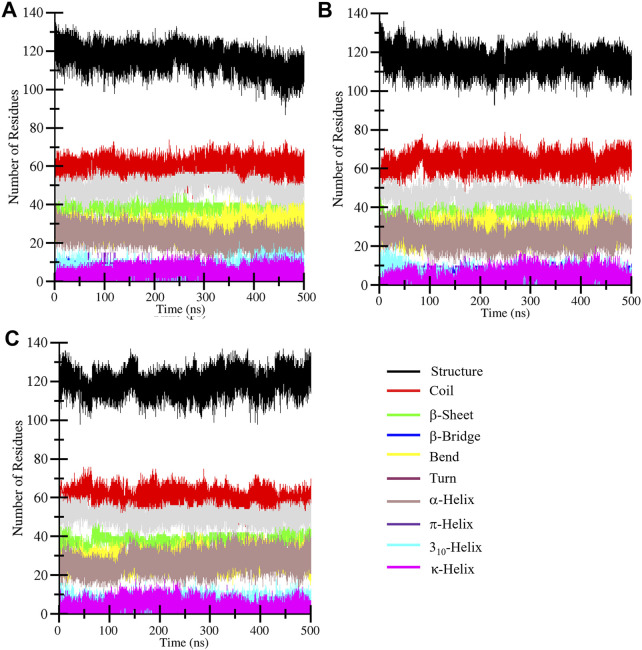
Secondary structure evolution of CathS and ligand-bound complexes. Secondary structure assignments of **(A)** CathS, **(B)** CathS-Q1N, and **(C)** CathS-Alectinib are shown across 500 ns simulations using DSSP. Minor fluctuations in α-helices, β-sheets, and turns are observed, but no major loss of secondary structure occurs, confirming the overall structural stability of CathS in both ligand-bound states.

Secondary structure analysis plays a critical role in assessing changes in the elements during simulations under the given factors. Secondary structure is composed of α-helix, β-sheet, β-bridge, and turns, which play a functional role in biological activity. The DSSP algorithm was employed to assess the secondary structure profile of the CathS after drug binding during a 500 MD run. Figure 7 displays different elements of secondary structure and their changes over time. The difference in residue involvement in secondary structure formation of CathS, CathS-Q1N, and CathS-Alectinib complex shows that CathS-Alectinib complex increased the number of residues, particularly at the end of the MD run, while a minor reduction was observed in the complex (Figures 7A–C). At some point, CathS-Alectinib complex also decreased residues’ participation in different elements of secondary structure, such as β-bridge and bend (Table 4). The result suggests that minor alterations were seen, which do not show any major impact on structural stability after drug binding.

**TABLE 4:** Alterations of the residues that participate in secondary structure formation calculated for CathS, CathS-Q1N, and CathS-Alectinib complex.

Systems	Structure	Coil	β-sheet	β-bridge	Bend	Turn	α-helix	Pi-helix	3_10_-helix	PPII-helix
CathS	116	59	38	5	28	23	49	1	5	4
CathS-Q1N	115	62	38	6	27	24	46	0.7	5	4
CathS-Alectinib	120	59	38	4	27	26	50	0.5	5	4

### 3.5 Principal component analysis

**FIGURE 8:**
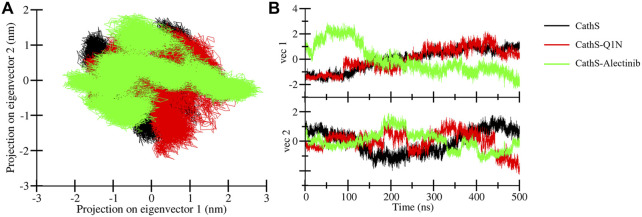
Principal component analysis plots. **(A)** PCA plot of CathS, CathS-Q1N, and CathS-Alectinib complex. **(B)** Time-dependent eigenvector representation of CathS, CathS-Q1N, and CathS-Alectinib complex.

PCA is a widely used statistical tool to determine the collective motion of the system (Sittel et al., 2014). PCA reduces the dimensionality of the datasets and provides critical information (Tharwat, 2016). We calculated the first two principal components for CathS, CathS-Q1N, and CathS-Alectinib complex because the first two principal components possess the most information and the largest variance in atomic motion (Figure 8). Figure 8A depicts two-dimensional PCA plots of CathS, CathS-Q1N, and CathS-Alectinib complex. A greater subspace covered by protein shows less stability, while the protein covered a lesser subspace is associated with higher stability. The reference CathS-Q1N complex overlapped with the CathS, while the CathS-Alectinib complex comparatively less vibrational area. The vibrational area covered by CathS at PC1 −1.94 nm to 1.5 nm, at PC2 −1.9 nm to 1.7 nm, CathS-Q1N at PC1 −1.8 nm to 2.1 nm, at PC2 −2.2 nm to 1.6 nm, and CathS-Alectinib complex at PC1 −2.3 nm to 2.8 at PC2 −1.4 nm to 1.9 nm. Moreover, time-based eigenvalue plots for CathS, CathS-Q1N, and CathS-Alectinib complex were also generated (Figure 8B). At PC1, the plot of CathS and CathS-Q1N complex was in increasing order after 200 ns, while the CathS-Alectinib complex plot was in a decreasing pattern after 200 ns simulation. At PC2, some random fluctuation was seen in all systems, and the CathS-Alectinib complex was comparatively downward mode at the end of the MD run. The findings suggest that the CathS-Alectinib complex was in stable form during the entire MD run.

### 3.6 Free energy landscapes

**FIGURE 9:**
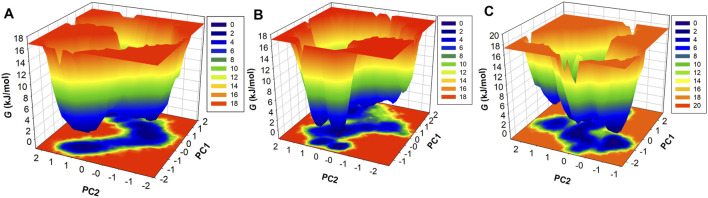
Free energy landscape (FEL) analysis of CathS and ligand-bound complexes. Three-dimensional FELs projected onto the first two principal components for **(A)** CathS, **(B)** CathS-Q1N, and **(C)** CathS-Alectinib. Color gradients range from red (high free energy, less stable states) to dark blue (low free energy, stable states). CathS alone exhibits a major deep basin near the native fold and additional shallow metastable states. The CathS-Q1N complex shows multiple shallow basins and sharper energy funnels, indicative of metastable intermediates. In contrast, the CathS-Alectinib complex displays a well-defined deep basin and connected funnels separated by intermediate barriers, reflecting a stable binding conformation with occasional transitions between substates.

FEL is used to measure the Gibbs free energy of the systems achieved during the simulation (Papaleo et al., 2009). The system that carries minimal energy corresponds to the stable or native fold of the protein. The FEL plot consisted of multiple colors, which indicates higher energy to lower energy, from red to dark blue (Figure 9). The CathS map had one dark blue basin, which is close to zero energy and tends to the native stable form; two light blue basins were also observed, which indicates different metastable states (Figure 9A). The CathS-Q1N complex map had one small-sized dark blue basin at a different place, which shows the meta-state of the complex (Figure 9B). CathS-Alectinib complex FEL map also carries one dark blue basin, which shows a different stable state of the complex, a smaller size blue basin closely associated with a highly stable form of the protein (Figure 9C). In the three three-dimensional FEL maps, the energy range of the CathS and CathS-Q1N complex was 0 kJ/mol to 18 kJ/mol, while the CathS-Alectinib complex shows 0 kJ/mol to 20 kJ/mol. The CathS map shows two dark blue basins, which relate to large energy barriers. CathS-Q1N complex map had multiple blue basins and energy funnels having sharp peaks, which indicate stability of the metastases. CathS-Alectinib complex map also available two dark blue basins and multiple energy funnels, which are connected through intermediate energy barriers. Each funnel reflects the fold of the metastases, which can easily adopt different conformations. Overall, FELs showed that the resulting CathS achieved its stable state when bound to docked ligands.

### 3.7 MM-PBSA analysis

The binding affinities of complexes CathS-Q1N and CathS-Alectinib were estimated using MM-PBSA calculations. This approach estimates the thermodynamic preference of the ligand–protein interaction, with more negative values of the binding free energy representing stronger and more stable associations. The overall binding energies were calculated by summing van der Waals, electrostatic, polar solvation, and non-polar solvation (estimated by SASA). Average binding free energies and their standard deviations are provided in Table 5 for each complex. For example, the estimated binding free energy Δ*G*_binding_ for the CathS–Alectinib complex is −20.16 ± 2.59 kcal/mol, which is substantially more negative than that for the CathS–Q1N reference complex (−6.17 ± 3.49 kcal/mol). This more negative Δ*G*_binding_ suggests a significantly stronger predicted affinity of Alectinib for CathS under the simulated conditions. Note that more negative binding free energy values indicate more favorable (stronger) binding in this computational framework. The outcome suggests that all depleted-energy components had positive effects on the stability of the ligand-CathS complexes. Van der Waals and electrostatic forces determined the predominant feature of the binding stability. The polar solvation energies were unfavorable as usually seen due to desolvation effects, but they were counterbalanced by favorable non-polar interactions through hydrophobic contacts. The relatively low standard deviations among the sampled frames suggest that the ligand–protein complexes remain stable during the trajectory. These MM-PBSA results agree with the earlier dynamics analyses, indicating that Alectinib may form a stable interaction with CathS. While these findings suggest potential utility of Alectinib as a CathS inhibitor, experimental validation is needed to confirm this prediction.

**TABLE 5:** Binding free energy parameters for CathS–ligand complexes calculated through the MM-PBSA approach.

Complex	Δ*G*_VDWAALS_	Δ*E*_EL_	Δ*E*_PB_	Δ*E*_NPOLAR_	Δ*G*_GAS_	Δ*G*_SOLV_	${∆G}_{\text{binding}\;\left(\text{kJ}/\text{mol}\right)}$
CathS-Q1N	−10.78	−3.41	9.62	−1.59	−14.19	8.03	−6.17 ± 3.49
CathS-Alectinib	−34.27	−3.52	21.47	−3.84	−37.79	17.63	−20.16 ± 2.59

## 4 Limitations and future directions

As with any study based solely on *in silico* approaches, the findings presented here should be interpreted with caution and viewed as hypothesis-generating rather than definitive. These results represent early-stage computational exploration and are not a substitute for biochemical or cellular validation and therefore have inherent boundaries that limit direct biological or clinical interpretation. The docking study and the MM-PBSA binding free energy calculations used in the virtual screening are approximations and depend on force field accuracy; absolute values can be imprecise and should be interpreted qualitatively. Moreover, the simulations did not account for the full complexity of a cellular environment. Alectinib’s primary target is ALK kinase, and its predicted activity on CathS is novel; potential off-target effects or differences in pharmacokinetics (distribution to pain-related tissues, etc.) are unknown. Future work should include experimental validation of CathS inhibition by Alectinib. Biochemical assays are needed to measure Alectinib’s affinity and inhibitory constant against CathS. Cellular studies in cancer and pain model systems would test whether Alectinib modulates CathS-related phenotypes. If CathS inhibition is confirmed, structure–activity relationship (SAR) studies could optimize Alectinib analogues for stronger CathS binding and reduced off-target effects. Additional computational work might include docking and simulations with multiple CathS conformations, free-energy calculations with enhanced sampling (e.g., metadynamics), and simulations with different force fields or solvent conditions to test robustness. Finally, pharmacokinetic and safety profiling should assess whether effective CathS inhibition by Alectinib can be achieved at tolerable doses. Addressing these points will clarify Alectinib’s potential as a CathS-targeting therapeutic.

## 5 Conclusion

Repurposing of FDA-approved drugs has indeed become a necessity in view of the growing burden of disease, including cancer. Virtual screening has now become an indispensable process in modern drug discovery, which is not only used for estimating the binding affinities and predicting the optimal binding orientations but also for the exploration of possible mechanisms of inhibition. Here, a structure-guided virtual screening strategy identified Alectinib, an FDA-approved ALK inhibitor, as a candidate repurposed CathS inhibitor for cancer and chronic pain through CathS inhibition. Alectinib was found to have good binding affinity, draggability, various anticancer properties, and antinociceptive activities. Interaction studies demonstrated consistent fusion with active-site amino acid residues (CYS139 and HIS278); this docking was reinforced by 500-ns of MD simulations and also ligand–protein interactions calculations by MM-PBSA with a minimum binding free energy of −20.16 ± 2.59 kcal/mol. Besides, PCA and FEL analyses verified the stability of the conformation of the CathS–Alectinib complex during the simulation. Together, these results indicate that Alectinib may have potential as a CathS inhibitor relevant to cancer and pain. These works, however, are computational predictions and have not been experimentally confirmed. Its potential off-target effects, pharmacokinetic profile in pain-specific tissues, and *in vivo* efficacy should be investigated. The inhibitory activity of Alectinib against CathS and its relevance as a therapeutic tool must be validated in experimental studies. In summary, the current investigation demonstrates the potential of *in silico* drug repurposing and characterizes Alectinib as a promising starting point for multi-indication CathS inhibition development.

## Data Availability

The original contributions presented in the study are included in the article/Supplementary Material, further inquiries can be directed to the corresponding author.
