# Supplementary material for: Computational drug repurposing reveals Alectinib as a potential lead targeting Cathepsin S for therapeutic developments against cancer and chronic pain
Source: Front Bioinform. 2025 Sep 24;5:1666573. doi: 10.3389/fbinf.2025.1666573 (PMC12504298; doi:10.3389/fbinf.2025.1666573)
Supplement: Supplementary file 1 [file Table1.docx]

**Table S1**: Drug profiles of the selected compounds from the docking screening.

| **S. No.** | **Drug molecule** | **Target** | **Properties** |
| --- | --- | --- | --- |
| 1. | Tasosartan | Angiotensin II type 1 receptor | Antihypertensive; angiotensin receptor blocker; vasodilation |
|  | Bagrosin | PDK3 | Anticancer; metabolic reprogramming blockade |
|  | Bisantrene | Topoisomerase II | Anticancer; DNA intercalator |
|  | Lumacaftor | CFTR (ΔF508) corrector | Cystic fibrosis therapy; protein‐folding chaperone |
|  | Tadalafil | Phosphodiesterase‑5 | Vasodilator; treatment of erectile dysfunction/pulmonary hypertension |
|  | Adapalene | Retinoic acid receptors (RARs) | Comedolytic; retinoid modulator; keratinization regulator |
|  | Eltrombopag | Thrombopoietin receptor (TPO‑R) | Thrombopoiesis stimulator; hematopoietic growth factor |
|  | Pimozide | Dopamine D₂ receptor | Antipsychotic; Ddopamine signaling blockade |
|  | Alprazolam | GABA_A receptor | Anxiolytic; central CNS depressant |
|  | Alectinib | ALK, RET | Anticancer, kinase inhibitor, downstream signaling blockade |
|  | Q1N | Cathepsin S | Protease inhibitor; immune modulation; anticancer |
